# Reconstruction of the Gene Regulatory Network Involved in the Sonic Hedgehog Pathway with a Potential Role in Early Development of the Mouse Brain

**DOI:** 10.1371/journal.pcbi.1003884

**Published:** 2014-10-09

**Authors:** Jinhua Liu, Xuelong Wang, Juan Li, Haifang Wang, Gang Wei, Jun Yan

**Affiliations:** CAS-MPG Partner Institute for Computational Biology, Shanghai Institutes of Biological Sciences, Chinese Academy of Sciences, Shanghai, China; Ottawa University, Canada

## Abstract

The Sonic hedgehog (Shh) signaling pathway is crucial for pattern formation in early central nervous system development. By systematically analyzing high-throughput in situ hybridization data of E11.5 mouse brain, we found that Shh and its receptor Ptch1 define two adjacent mutually exclusive gene expression domains: Shh^+^Ptch1^−^ and Shh^−^Ptch1^+^. These two domains are associated respectively with Foxa2 and Gata3, two transcription factors that play key roles in specifying them. Gata3 ChIP-seq experiments and RNA-seq assays on Gata3-knockdown cells revealed that Gata3 up-regulates the genes that are enriched in the Shh^−^Ptch1^+^ domain. Important Gata3 targets include *Slit2* and *Slit3*, which are involved in the process of axon guidance, as well as *Slc18a1*, *Th* and *Qdpr*, which are associated with neurotransmitter synthesis and release. By contrast, Foxa2 both up-regulates the genes expressed in the Shh^+^Ptch1^−^ domain and down-regulates the genes characteristic of the Shh^−^Ptch1^+^ domain. From these and other data, we were able to reconstruct a gene regulatory network governing both domains. Our work provides the first genome-wide characterization of the gene regulatory network involved in the Shh pathway that underlies pattern formation in the early mouse brain.

## Author Summary

Recent large-scale projects of high-throughput in situ hybridization (ISH) have generated a wealth of spatiotemporal information on gene expression patterns in the early mouse brain. We have developed a computational approach that combines publicly available high-throughput ISH data with our own experimental data to investigate gene regulation, involving signal molecules and transcription factors (TFs), during early brain development. The analysis indicates that two key TFs, Foxa2 and Gata3, play antagonizing roles in the formation of two mutually exclusive domains established by the Sonic hedgehog signaling pathway in the developing mouse brain. Further ChIP-seq and RNA-seq experiments support this hypothesis and have identified novel target genes of Gata3, including the axon guidance regulators *Slit2* and *Slit3* as well as three neurotransmitter-associated genes, *Slc18a1*, *Th* and *Qdpr*. The findings have allowed us to reconstruct the gene regulatory network brought into play by the Sonic hedgehog pathway that mediates early mouse brain development.

## Introduction

Pattern formation in early animal development is controlled by signal transduction cascades, in which transcription factors (TFs) play crucially important roles as downstream effectors. The signal transduction cascades together with the gene regulatory networks they activate determine the temporal and spatial expression of a wide range of genes for the specification of regions and differentiation of cells [1].

Sonic hedgehog (Shh) is a classical signal molecule required for pattern formation in many aspects of animal development, not least in neural development. In the central nervous system (CNS), depending on the graded Shh concentration along the dorsal-ventral axis in the mouse ventral neural tube, particular TFs are activated in different regions, resulting in specification of these regions [2]–[5]. The Shh signaling pathway is itself activated when Shh binds to its receptor Ptch1, which, without the ligand, inhibits the cell membrane protein Smo. Shh binding removes the inhibition on Smo and triggers the activation of three GLI family TFs, Gli1, Gli2 and Gli3, which further activate or inhibit specific TFs to determine regional cell fate. Identifying those downstream TFs and how they work is a central task in the elucidation of early CNS development.

Several recent studies using high-throughput in situ hybridization (ISH) have provided a rich harvest of information on spatio-temporal gene expression in early mouse development. The data are available in databases such as GenePaint [6], Eurexpress [7] and Allen Brain Atlas (ABA) [8]. GenePaint and Eurexpress have focused on whole mouse embryos at the E14.5 stage and covering almost the entire set of known mouse genes. In contrast, ABA (http://developingmouse.brain-map.org) recently offered manually annotated ISH data for the developing mouse brain from three developmental stages: E11.5, E13.5 and E15.5. It includes information about expression intensity, density and pattern for more than 2000 genes, many of which are TFs and key genes in early brain development. Because ISH data contain high-resolution spatial information on gene expression, they are invaluable for in-depth study of gene regulation in pattern formation during early development. For example, Visel et al. showed that it is possible to identify the probable targets of Pax6, a key TF in early mouse brain, by the clustering of co-expressed genes using E14.5 ISH data [9].

However, co-expression of genes does not guarantee that they are directly co-regulated by the same TF. Furthermore, developmental genes in animals are often regulated by a combination of TFs acting through cis-regulatory modules [10]. Therefore, high-throughput ISH data has to be integrated with direct gene regulatory data such as genome-wide ChIP-seq data to delineate the specific regulatory mechanisms underlying particular developmental processes. Such an approach would be very useful in elucidating the genetic networks involved in early brain development.

Gata3 and Foxa2 are two key TFs implicated in early animal development, including early brain development. Gata3 is a member of the GATA family, consisting of Gata1-6, among which only Gata2 and Gata3 have been reported to be expressed in the CNS [11]. Mice homozygous for a Gata3 null mutation were found to have serious malformations of the embryonic brain, revealing its essentiality for that stage [12]. Furthermore, continuous expression of Gata3 in the brain from early embryo to adulthood suggests that it is important for the maintenance of brain functions beyond early development [13]. Recent genome-wide studies of Gata3 have mainly focused on the molecular mechanisms underlying its critical roles in T cells [14] and breast cancer [15]. In breast cancer, Gata3 has been shown to function as a “pioneer factor” to help open up condensed chromatin and recruit other TFs. However, no genome-wide study on Gata3 in the CNS has been conducted so far.

Foxa family TFs including Foxa1, Foxa2 and Foxa3 are involved in development, organogenesis [16] and metabolism [17]. Similarly to Gata3, there is increasing evidence that they also play crucial roles as pioneer factors [18]. Unlike Gata3, however, the role of Foxa2 in brain development has been better studied. Notochord-secreting Shh required for patterning of the neural tube fails to form when Foxa2 is mutated, hindering the entire subsequent developmental process [19]. Genome-wide ChIP-seq analysis of Foxa2 targets in midbrain dopaminergic neuron (mDA) progenitors revealed that Foxa2 directly regulates key genes in the Shh signaling pathway and that Foxa2 promotes gene expression in the floor plate while repressing the genes normally expressed and required in the ventro-lateral region of midbrain [20].

In this study, we investigated gene regulation in the Shh signal transduction cascade in early developing mouse brain, focusing on Gata3 and Foxa2 as two putative key TFs. We have found that they demarcate two mutually exclusive domains in the early mouse brain coinciding with two domains defined by the reciprocal expression patterns of Shh and its receptor Ptch1. These will be designated as the Shh^+^Ptch1^−^ and Shh^−^Ptch1^+^ domains. To understand the molecular functions of Gata3 in the early mouse brain, we used PC12 cell line established from rat adrenal medulla pheochromocytoma to mimic the Gata3-expressed domain in early mouse brain. We performed Gata3 ChIP-seq in PC12 cells and RNA-seq experiments in Gata3 siRNA knockdown cells. We found that Gata3 target genes that are down-regulated by Gata3 siRNA knockdown were enriched in the Gata3-expressed domain. By contrast, Foxa2 target genes were enriched in both Foxa2- and Gata3-expressed domains. These results suggested that the fates of the two domains were controlled by distinct regulatory mechanisms directed by Gata3 and Foxa2. The interaction between these two domains was transmitted via the Shh signaling pathway. In addition, we identified, amongst Gata3 target genes, *Slit2* and *Slit3*, which are involved in axon guidance, as well as *Slc18a1*, *Th* and *Qdpr*, which function in neurotransmitter synthesis and release. From these findings and ChIP-seq data, we were able to reconstruct a gene regulatory network for the genes in Shh^+^Ptch1^−^ and Shh^−^Ptch1^+^ domains. Our study expands current knowledge of the Shh pathway and sheds new light on the gene regulatory mechanisms controlling cell fates in the early mouse brain.

## Materials and Methods

### Identification of Shh^+^Ptch1^−^-pattern and Shh^−^Ptch1^+^-pattern genes

We used ISH data of developing mouse brain at E11.5 stage from the ABA database. The data consists of more than 2000 genes manually annotated by experts for the ISH image series. Gene expression properties were characterized by utilizing three metrics: intensity (Undetected, Low, Medium and High), density (Undetected, Low, Medium and High) and pattern (Undetected, Full, Regional and Gradient). In our study, to convert the textual annotation to numerical data, we used intensity as the metric and treated “Undetected” as 0 and “Low, Medium and High” as 1 for our downstream analysis. Based on this gene expression data of E11.5 mouse brain, we observed that the expression patterns of Shh and its receptor Ptch1 were obviously stratified along the ventral-dorsal axis. From this observation, we defined the Shh^+^Ptch1^−^ domain as containing 20 brain sub-regions in the anatomical map provided by ABA, and the Shh^−^Ptch1^+^ domain, which we found contains 30 brain sub-regions (Figure S1). Next, Fisher's exact test was used to identify genes expressed exclusively in the Shh^+^Ptch1^−^ domain (*P* value <0.0001, odds ratio >1, expressed in more than 10 Shh^+^Ptch1^−^ sub-regions) and those in the Shh^−^Ptch1^+^ domain (*P* value <0.0001, odds ratio <1, expressed in more than 15 Shh^−^Ptch1^+^ sub-regions). These two sets were accordingly defined, respectively, as Shh^+^Ptch1^−^-pattern genes and Shh^−^Ptch1^+^-pattern genes. A heatmap containing these two types of genes was generated by the R program (http://www.r-project.org) and is shown in supplementary material Figure S2.

### Promoter analysis

The gene annotations and repeat-masked genome sequences for six mammalian species including human, marmoset, mouse, rat, cow, pig were downloaded from ENSEMBL (version 62). Promoter sequences defined as the region upstream 1000 bp to downstream 200 bp from transcriptional start site (TSS) were extracted using Perl Script from each species. For each mouse gene, we obtained their orthologous gene information in the other five mammalian species using ENSEMBL homologs data (version 62). Promoter analysis was performed based on Pscan program [21], by which we can obtain the enriched transcription factor (TF) binding motifs in each set of promoters of orthologous genes. The relationships between TF binding motifs and TFs were obtained from TRANSFAC [22]. In this study, TF motif-target relationships were determined by selecting TF motifs with the criteria that enrichment *P* value less than 0.005 and the rank is at least top 20. Motif enrichment in the promoter sequences of Shh^+^Ptch1^−^- and Shh^−^Ptch1^+^-pattern genes were performed using Pscan solely on mouse genes. Enriched TF groups were selected with *P* values less than 0.005.

### Functional analysis

The functional analysis of gene sets based on gene ontology (GO) resources was performed using GOToolBox program (http://genome.crg.es/GOToolBox) with “Mouse Genome Informatics (MGI)” and “Rat Genome Database (RGD)” as the corresponding annotations respectively for distinct sets of genes. Results are supplied in Table S1. The statistical significance of enrichment between gene group of interest and background gene group was calculated by applying the one-sided Fisher's exact test.

### Cell culture

PC12 cells were plated on a Poly-L-lysine-coated dishes (Corning) and maintained in DMEM/F12 (Invitrogen) with 5% FBS (Biochrom), 5% horse serum (Gibco) and 1% penicillin/streptomycin at 37°C in 5% CO2.

### ChIP and ChIP-seq

ChIP assays were carried out using materials from PC12 cells and performed as described previously [23]. Briefly, cells were cross-linked with formaldehyde and sonicated to generate chromatin fragments size-enriched to between 200–600 bp. Antibody against GATA3 (558686, BD Pharmingen™) was used. Chromatin from 20 million cells was used for each ChIP experiment, which yielded approximately 10 ng of DNA. As input, 2% of sonicated chromatin was treated with proteinase K at 50°C for 2 hr and purified using the QIAquick PCR Purification Kit (Qiagen Cat # 28106). Both input DNA and ChIP DNA fragments were blunt-ended, ligated to the Illumina adaptors, and sequenced with the Illumina Hiseq 2000.

### ChIP-seq data analysis

Sequencing reads of ChIP-seq were mapped to the rat genome (Baylor 3.4/rn4) using Bowtie (version 1.0.0) [24], with the setting that sequence alignments can have no more than 3 mismatches. Then MACS (Model-based Analysis of ChIP-seq; version 1.4.2) [25] was used to identify Gata3 binding regions and peak summits which were further annotated by using CEAS [26]. Two tools in Cistrome were deployed to calculate the correlation coefficient for our biological replicates and the PhastCons scores [27]. De novo motif analysis was performed using MEME-ChIP version 4.9.0 [28] after masking query sequences using RepeatMasker (http://www.repeatmasker.org/). A gene was defined to be the target gene containing a binding site if this site is located between 10 kb upstream of transcription start site (TSS) and 3 kb downstream of transcription end site (TES) of this gene with the exception that the binding site on *Th* was found when we extended its promoter region to17,491 bp upstream of TSS. The MACS output file about binding sites, together with the associated target genes, is provided in Table S4.

### siRNA transfections and RNA-seq

We used two different custom-made siRNAs against Gata3. siGATA3-1 (sense 5′-GUACUACAAACUCCACAAUTT-3′ and antisense 5′-AUUGUGGAGUUU GUAGUACTT-3′), siGATA3-2 (sense 5′-CCGUAAGAUGUCUAGCAAATT-3′ and antisense 5′-UUUGCUAGACAUCUUACGGTT3′) and negative control (sense 5′-UUCUCCGAACGUGUCACGUTT-3′ and antisense 5′-ACGUGAC ACGUUCGGAGAATT) were obtained from GenePharma (Shanghai). All siRNA experiments were conducted at a final concentration of 50 nM. Transfections were conducted using Lipofectamine RNAiMAX (Invitrogen). Total RNA was isolated from cells using Trizol (Invitrogen). Purified mRNA was used to prepare the cDNA library as per the manufacturer's instructions. The short cDNA fragments were ligated to the Illumina sequencing adaptors and sequenced with the Illumina Hiseq 2000.

### Real-Time PCR

Total RNA was isolated from cells to synthesize cDNA with SuperScript II Reverse Transcriptase (Invitrogen). qRT-PCR amplification mixtures (20 µl) contained 3 µl water, 1 µM forward and reverse primer, 10 µl LightCycler 480 DNA SYBR Green I Master Mix buffer and 5 µl template cDNA. All reactions were run on LightCycler 480 (Roche).

### RNA-seq data analysis

All sequencing reads of RNA-seq were mapped to the rat genome using TopHat with default settings (http://tophat.cbcb.umd.edu/; version 2.0.7) [29]. The output data were analyzed by Cuffdiff to identify differentially expressed genes [30]. The results were filtered by the criteria: “status” = OK and “*P* value”<0.05. Our ChIP-seq and RNA-seq data were submitted to ArrayExpress database with accession number: E-MTAB-2008.

### Microarray data analysis

The original CEL files of GSE42565 from the Shh stimulation experiment [31] and GSE15942 performed in PC12 cells [32] were downloaded from Gene Expression Omnibus (GEO). The method “RMA” from R package “affy” was used to normalize the raw data. For GSE42565, student's t-test was used to identify differentially expressed genes responding to the Shh stimulation. 1677 genes were selected as the downstream genes of Shh on the basis that they had a *P* value <0.05.

## Results

### Restricted expression patterns of *Shh* and *Ptch1* define two adjacent early brain domains

**Figure 1:**
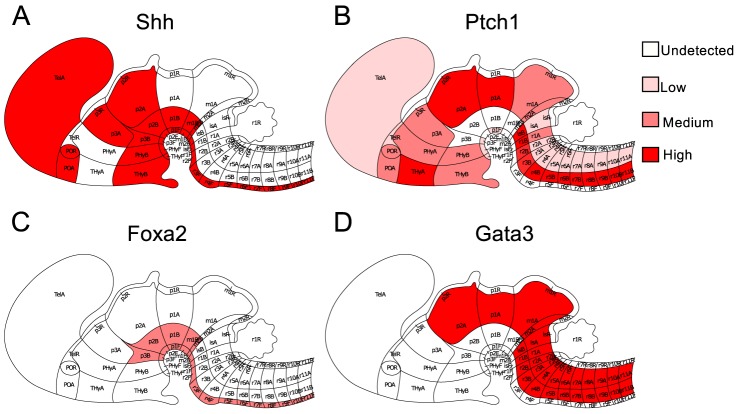
The expression patterns of *Shh*, *Ptch1*, *Foxa2* and *Gata3* in E11.5 mouse brain from ABA. Red colors ranging from light to dark indicate the gene expression intensities ranging from low to high. The expression patterns of *Shh* and *Ptch1* defined two adjacent non-overlapping brain domains: Shh^+^Ptch1^−^ domain and Shh^−^Ptch1^+^ domain as shown in supplementary material Figure S1. The anatomical maps were downloaded from ABA and the expression patterns were mapped manually according to the ISH annotation file.

In this study, the expression patterns of 2074 genes, manually annotated based on ISH images for 78 regions in E11.5 mouse brain, were downloaded from Allen Brain Atlas. We converted the textual annotation of ISH data to binary gene expression data of 0 and 1 (Materials and Methods). We observed that the genes coding for key signaling molecules, such as *Fgf8* expressed in 9 regions, *Shh* in 31regions, *Notch2* in 24 regions, *Bmp1* in 12 regions and *Bmp4* in 3 regions as well as critical developmental genes including *En1* in 25 regions, *En2* in 12 regions, *Hes3* in 3 regions and *Otx2* in 34 regions, showed restricted expression patterns at the E11.5 stage. In particular, we found that the gene expression patterns of Shh and its receptor Ptch1 were clearly segregated along the ventral-dorsal axis, especially in the regions from midbrain to hindbrain. *Shh* was highly expressed in the ventral brain region while *Ptch1* was expressed just above the *Shh*-expressed region. *Shh* occupied the entire floor plate while *Ptch1* occupied the whole alar plate and most of the basal plate (Figure 1A and 1B). We used the expression patterns of *Shh* and *Ptch1* to define two adjacent non-overlapping brain domains: a Shh^+^Ptch1^−^ domain where *Shh* is expressed but *Ptch1* is not expressed and a Shh^−^Ptch1^+^ domain, defined by the reciprocal pattern, where *Shh* is not expressed but *Ptch1* is expressed (Figure S1).

Next, to identify the factors controlling the specification of these two domains, we searched for the genes specifically expressed in the Shh^+^Ptch1^−^ and Shh^−^Ptch1^+^ domains respectively. We identified 45 Shh^+^Ptch1^−^-pattern genes and 337 Shh^−^Ptch1^+^-pattern genes using Fisher's exact test (P<0.0001) (Figure S2). We then cross-compared these two groups of genes with the genes specifically expressed in midbrain floor plate (FP) and ventral-lateral region (VL) of neural tissues obtained from an independent microarray study [33]. We found that the Shh^+^Ptch1^−^-pattern genes were significantly enriched among the FP genes while the Shh^−^Ptch1^+^-pattern genes were enriched among the VL genes (Figure S3A and S3B). The consistency between the two datasets supported our method of defining the Shh^+^Ptch1^−^-pattern and Shh^−^Ptch1^+^-pattern genes based on ISH data. Gene ontology (GO) enrichment analysis revealed that biological processes of system development, anatomical structure development and regulation of transcription were significantly enriched in both Shh^+^Ptch1^−^-pattern and Shh^−^Ptch1^+^-pattern genes, which highlighted their importance in early brain development (Table S1, Figure S3C and S3D).

### *Gata3* and *Foxa2* expression patterns are mutually exclusive in Shh^−^Ptch1^+^ and Shh^+^Ptch1^−^ domains

To discover the potential transcriptional regulators for Shh^+^Ptch1^−^- and Shh^−^Ptch1^+^-pattern genes, we conducted promoter analysis for these two groups of genes. Motif enrichment analysis showed that known TF binding motifs for GATA and GLI family TFs were significantly enriched in the promoters of Shh^−^Ptch1^+^-pattern genes (p = 5.95e–05 for GATA motif, p = 6.82e–06 for GLI motif) but not in Shh^+^Ptch1^−^-pattern genes (Table S2), indicating the importance of these two families of TFs in controlling the specification of the Shh^−^Ptch1^+^ domain. Interestingly, GLI family TFs Gli1 and Gli2, the downstream transducers of the Shh signaling pathway, are expressed in the Shh^−^Ptch1^+^ domain but not in the Shh^+^Ptch1^−^ domain. Furthermore, our promoter analysis predicted that both Gli1 and Gli2 directly target the GATA family member TF *Gata3*, which is known to be a pioneer factor and strictly expressed in the Shh^−^Ptch1^+^ domain. Therefore, it is likely that Shh secreted in the Shh^+^Ptch1^−^ domain diffuses to the neighboring Shh^−^Ptch1^+^domain to exert its influence via the transcriptional activation of Gata3. In other words, Gata3 may determine the specification of the Shh^−^Ptch1^+^ domain via the Shh signaling pathway. In contrast, in the Shh^+^Ptch1^−^ domain, among all of the eight Shh^+^Ptch1^−^-pattern TFs annotated by ABA, Foxa1 and Foxa2 have been shown to function as master regulators to specify the identity of ventral midbrain progenitor cells by regulating Shh signaling [34]. As shown in a published Foxa2 ChIP-seq dataset [20], Twenty four out of the total of 45 identified Shh^+^Ptch1^−^-pattern genes, including *Shh*, were targeted by Foxa2. There is evidence that *Shh* is activated by Foxa2 while its downstream effectors, *Ptch1*, *Gli1*, *Gli2* and *Gli3* are all repressed by Foxa2 [20], [34]. This would explain the absence of *Ptch1*, *Gli1*, *Gli2* and *Gli3* expression in the Shh^+^Ptch1^−^ domain and suggests that Foxa2 plays a key role in determining the fate of the ventral Shh^+^Ptch1^−^ domain. These observations led us to propose that the Shh signaling pathway affects the pattern formation of the Shh^+^Ptch1^−^ and the Shh^−^Ptch1^+^ domains in E11.5 mouse brain along the ventral-dorsal axis via the mutually exclusive expression of Foxa2 and Gata3.

In total, we found 8 and 147 TF genes in the Shh^+^Ptch1^−^-pattern and Shh^−^Ptch1^+^-pattern, respectively. Amongst the Shh^−^Ptch1^+^-pattern TFs, critical developmental genes such as *Pax6*, *Pax3*, *Lhx1*, *Irx3*, *Isl*, *Ascl1* and *Gata3* were found. For these two groups of TFs, we were able to predict their regulatory targets within the two domain patterns by promoter analysis. Some known regulatory relationships, such as Foxa1 targeting *Foxa2* and Gli1/2 targeting *Ptch1*, were correctly recapitulated by our promoter analysis [16]. Five out of eight predicted targets of Foxa2 including *Nfib, Aff3, Foxa2, Foxq1* and *Nfia* were supported by the Foxa2 ChIP-seq data [20]. Notably, Foxa2 ChIP-seq data showed that Foxa2 targets*Gata3* and our promoter analysis predicted that Gata3 targets *Foxa2*. Together with the non-overlapping expression patterns of *Foxa2* and *Gata3* (Figure 1C and 1D), it seems to suggest a potential mutual inhibitory relationship between Foxa2 and Gata3.

### Genome-wide ChIP-seq analysis of Gata3 in PC12 cells

**Figure 2:**
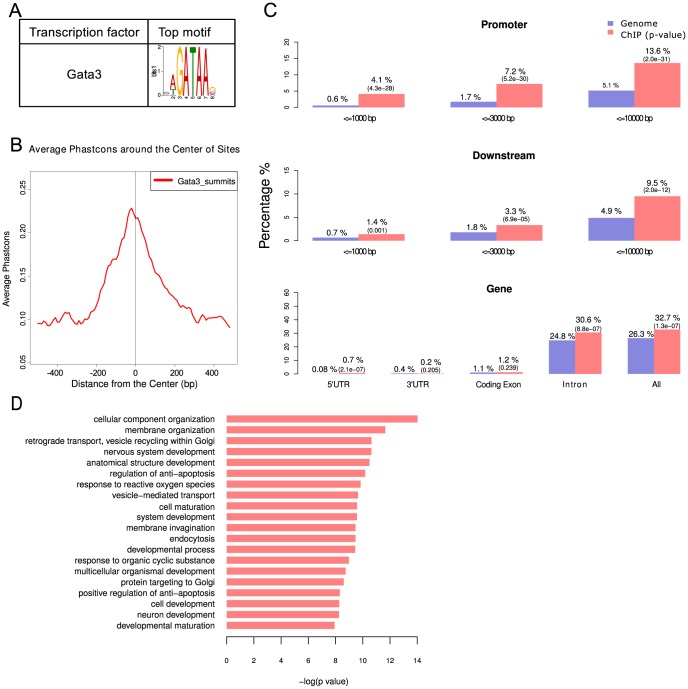
Genome-wide characterization of Gata3 binding sites in ChIP-seq. (A) Top binding motif identified by MEME-ChIP. (B) Conservation plot of Gata3 binding sites in vertebrate species. (C) The genome-wide distribution of Gata3-binding sites. (D) The enriched biological processes in Gata3-binding targets revealed by GO analysis.

The role of Foxa2 in regulating the expression of Shh and other genes expressed in the Shh^+^Ptch1^−^ domain has been previously characterized [20]. Here, however, we investigated the functional role of Gata3 in mediating the Shh signaling pathway in the Shh^−^Ptch1^+^domain. To this end, we sought a proper cell line that can mimic the gene expression pattern of this domain. We therefore analyzed the expression of Shh^−^Ptch1^+^-pattern genes in published microarray data available in Gene Expression Omnibus (GEO) for neuron-like cell lines, including PC12, neuro2a and N1E cells. Shh^−^Ptch1^+^-pattern genes, when compared to other genes annotated by ABA, were only found to be significantly enriched among the highly expressed genes of the PC12 cells (Fisher's exact test, *P* value  = 0.00007) but not in neuro2a and N1E cells. In particular, according to the microarray data as well as our Real-time PCR assay, *Gata3* has high expression in PC12 cells while *Foxa2* is not expressed (Table S3). PC12 cells are able to synthesize noradrenaline [35] and have the properties of neurons in that their exposure to Neuron Growth Factor (NGF) causes them to stop dividing and begin to grow neurites similar to those of sympathetic neurons. This neuron-like character makes this cell line a versatile model system for researches in neurobiology and neurochemistry [35]. Therefore, we selected PC12 cells to perform ChIP-seq [36] analysis for Gata3 and specifically to identify its target genes. Our ChIP-seq experiments included two biological replicates for ChIP and input materials respectively. The high correlation (Pearson's r = 0.97) between the two ChIP replicates suggested that our ChIP experiments were highly reproducible. After mapping all sequencing reads to the rat genome (rn4), we used the MACS program for peak calling, which yielded 1296 peaks with a default *P* value cutoff (Table S4). De novo motif analysis of these binding regions by MEME-ChIP revealed a significantly enriched Gata3 motif (Figure 2A). The elevated average phastcon scores around the center of Gata3 binding sites suggested that Gata3 binding sites were more conserved compared with the neighboring regions, an indication of functional binding sites (Figure 2B). We used the CEAS program to examine the distribution of Gata3 binding sites across the genome. We found that Gata3 binding sites were significantly enriched in the promoter regions with respect to the whole genome, i.e. 4.1% of ChIP regions fell within 1000 bp, 7.2% within 3000 bp and 13.6% within 10000 bp upstream of the transcription start site (TSS) of different genes. Furthermore, 32.7% of Gata3 binding sites were located in the gene bodies compared to 26.3% in the genome background. Among the binding sites in the gene bodies, 30.6% were within introns, 0.2% within the 3′UTRs and 0.7% within the 5′UTRs (Figure 2C).

**Figure 3:**
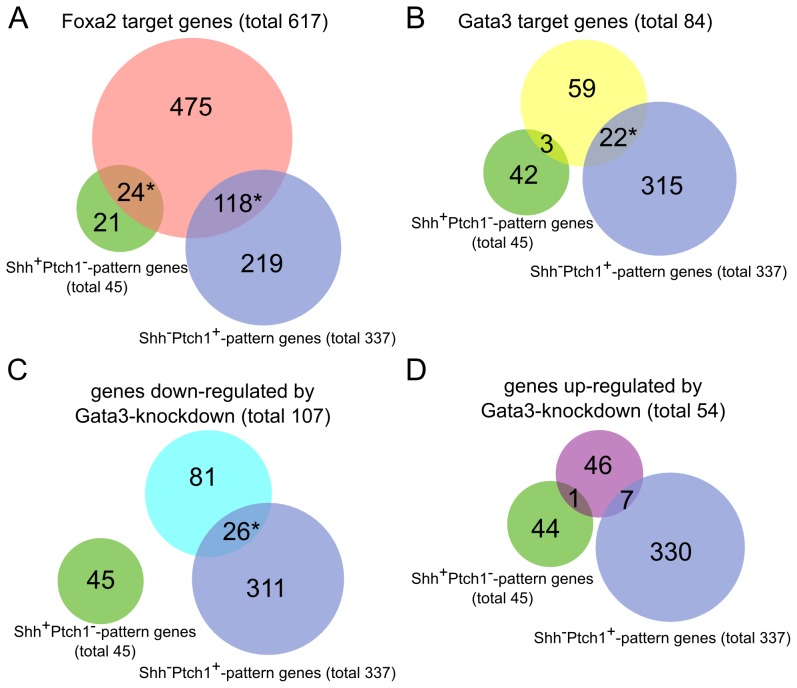
The relationship of Gata3 and Foxa2 targets with Shh^+^Ptch1^−^-/Shh^−^Ptch1^+^-pattern genes. (A) The overlaps between Foxa2 targets in ChIP-seq with ABA ISH annotations and Shh^+^Ptch1^−^-/Shh^−^Ptch1^+^-pattern genes. (B) The overlaps between 84 Gata3 targets in ChIP-seq with ABA ISH annotations and Shh^+^Ptch1^−^-/Shh^−^Ptch1^+^-pattern genes. (C–D) The overlaps between genes with ABA ISH annotations down- or up-regulated by Gata3-knockdown and Shh^+^Ptch1^−^-/Shh^−^Ptch1^+^-pattern genes. (* indicates the statistical significance of the overlap, *P*<0.05, in Fisher's exact test).

Using the gene annotation data of the rat rn4 genome downloaded from UCSC, we obtained 683 Gata3 target genes in PC12 cells (Table S4). GO analysis showed that these Gata3 targets were involved in biological processes such as nervous system development, cell differentiation, and cell maturation (Figure 2D). While Foxa2 targets were significantly enriched in genes in both the Shh^+^Ptch1^−^- and Shh^−^Ptch1^+^-patterns, Gata3 targets identified in our study were only enriched in Shh^−^Ptch1^+^-pattern genes but not in Shh^+^Ptch1^−^-pattern genes, indicating that Gata3 mainly influences the Shh^−^Ptch1^+^domain (Figure 3A and 3B). The genes of eight Shh^−^Ptch1^+^-pattern TFs, including Abl1, Cebpe, Gata2, Isl2, Myt1l, Nfib, Pou2f2 and Sox12, were targeted by Gata3, as shown by our ChIP-seq experiment.

### Gata3 is involved in the Shh signaling pathway

**Figure 4:**
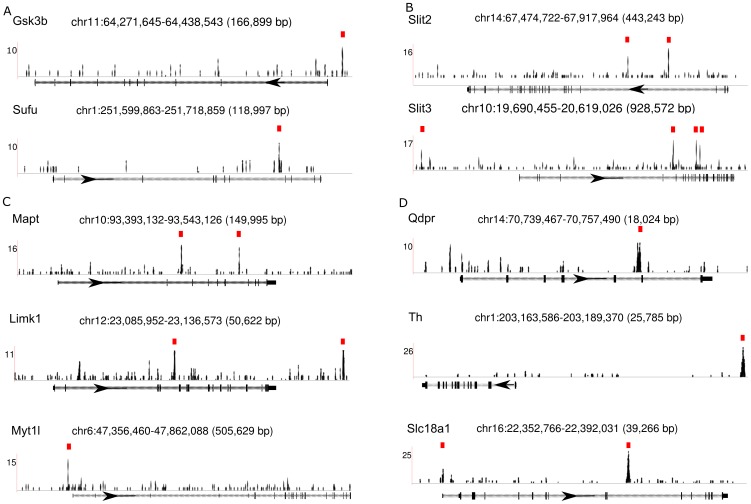
Illustration of Gata3 ChIP-seq binding sites on the selected genes. Red boxes indicate the binding peak locations called by MACS program. (A) *Sufu* and *Gsk3b* in the Shh signaling pathway. (B) *Slit2/3* involved in axon guidance. (C) *Nfasc, Mapt* and *Limk1* regulating brain development. (D) *Qdpr, Th* and *Slc18a1* involved in neurotransmitter synthesis and release.

Among Gata3 targets identified by ChIP-seq, we found two known regulators of the Shh signaling pathway, *Sufu* and *Gsk3b* (Figure 4A). Previous studies have shown that Sufu negatively regulates Shh signaling by direct interaction with Gli1 protein [37] and that Sufu is involved in Gli3 phosphorylation mediated by Gsk3b to induce the repression of Shh downstream genes [38]. To further investigate the involvement of Gata3 in the Shh signaling pathway, we systematically searched for Shh downstream genes by analyzing a published microarray dataset (GSE42565) on Shh stimulation in in vitro neural progenitors [31]. We found 74 Gata3 ChIP-seq target genes among the downstream genes of Shh. Among them, 45 genes including *Slit2* and *Slit3* were up-regulated by Shh stimulation (Figure 4B) and 29 genes were down-regulated by Shh (Table S5). Sixteen out of the 45 Gata3 target genes up-regulated by Shh were annotated by ABA in E11.5 ISH data. Eight of them were Shh^−^Ptch1^+^-pattern genes including *Cotl1*, *Foxn3*, *Klhl29*, *Limk1*, *Mapt*, *Myt1l*, *Nfasc* and *Scg3* (Figure 4C). Nfasc is well-known as a cell adhesion molecule important for cell-cell communication and neurite outgrowth. Nfasc also influences cell differentiation and maintenance in the brain but the signaling pathways upstream of Nfasc in the nervous system are unclear [39]. Two of the genes in this set, Mapt and Limk1, are known to be essential for brain development. A Mapt mutation is associated with neurodegenerative disorders such as Alzheimer disease [40], while the brain-specific Limk1 is implicated in axonal elongation [41]. Our study indicated that the specification of these genes in the Shh^−^Ptch1^+^ domain is likely due to Gata3 regulation in the Shh signaling pathway.

### Gata3 influences neurotransmitter synthesis and release

Gata3 is known to control the synthesis of noradrenaline and serotonin [42]. The ISH data for *Th*, *Ddc*, and *Dbh*, which are involved in noradrenaline synthesis, and *Tph2*, which is involved in serotonin synthesis, support the idea that the Shh^−^Ptch1^+^domain includes brain regions that eventually develop into noradrenergic and serotonergic neurons. Previous work has shown that a mutation in Gata3 reduced *Th* expression [43]. In our ChIP-seq experiments, we found Gata3 binding sites located in the potential promoter region of *Th* (Figure 4D). Furthermore, we found that Gata3 regulated two other neurotransmitter-associated genes, *Qdpr* and *Slc18a1* (Figure 4D). Qdpr is an enzyme involved in biosynthesis of tetrahydrobiopterin biosynthesis, which functions as a coenzyme in the reaction converting tyrosine to L-DOPA catalyzed by Th. L-DOPA can further lead to the formation of neurotransmitters including dopamine, noradrenaline, and adrenaline.Slc18a1 is a vesicular transporter that transports neurotransmitters including dopamine, noradrenaline, adrenaline and serotonin into synaptic vesicles and which thus plays an important role in neurotransmitter release. Functional disruption of Slc18a1 leads to neuropsychiatric diseases resulting from disorders of the corresponding neurotransmitter systems [44]. Our discovery that Gata3 targets the promoters of *Qdpr* and *Slc18a1* further supports Gata3's important role in neurotransmitter synthesis and release.

### Gata3 regulates SLIT/ROBO system

To further uncover the functional roles of Gata3, we applied siRNAs to knockdown Gata3 in PC12 cells. RNA-seq was performed in Gata3 siRNA-knockdown PC12 cells. Comparing our RNA-seq data with a published microarray data in wild-type PC12 cells (GSE accession: GSE15942) showed that they are highly correlated (Spearman's Rho  = 0.77, *P* value <2.2e–16). The gene expression values of two independent Gata3-knockdown samples with knockdown efficiencies of 50% and 51% respectively were also highly correlated (Pearson's r = 0.997; *P* value <2.2e–16). We integrated these results and obtained 1,121 differentially expressed genes compared to wild-type PC12 cells. Among them, 731 that were down-regulated by Gata3-knockdown, including Gata3 itself, were enriched in Shh^−^Ptch1^+^-pattern genes. By contrast, 390 up-regulated genes were not enriched in either the Shh^−^Ptch1^+^ or Shh^+^Ptch1^−^ patterns (Table S6, Figure 3C and 3D). This result supports our hypothesis that Gata3 preferentially up-regulates Shh^−^Ptch1^+^-pattern genes.

We then integrated the results of Gata3 ChIP-seq and Gata3 knockdown RNA-seq. Seventy seven (77) differentially expressed genes in RNA-seq assays were directly targeted by Gata3. The RNA-seq analysis revealed that *Slc18a1* was up-regulated after Gata3 knockdown. Notably, the expression of two Gata3-targeted genes from ChIP-seq, *Slit2* and *Slit3* (Figure 4B), together with *Robo1*, were all down-regulated after Gata3-knockdown. Other identified genes were further validated by our Real-time PCR analysis (Table S3). SLIT/ROBO, functioning as a ligand/receptor signaling system, is involved in axon guidance and neuronal migration in the CNS. Its special function in regulating axons to project across the midline has attracted a lot of attention [45]. Furthermore, recent studies have suggested that the Slit2/Robo1 signaling might be enlisted for treating glioma because it can inhibit glioma cell migration [46]. Earlier microarray analysis of Shh-induced expression also suggested that *Slit2/3* were downstream genes of the Shh pathway. Altogether, our results demonstrate that the SLIT/ROBO system was activated by Shh through the direct regulation of Gata3 in the Shh^−^Ptch1^+^ domain.

### Gene regulatory network in Shh^+^Ptch1^−^ and Shh^−^Ptch1^+^ domains

We next reconstructed a gene regulatory network downstream of the Shh signaling pathway in early mouse brain. We downloaded all suitable ChIP-seq data from Gene Expression Omnibus (GEO) database or published papers [47]–[50] for Shh^+^Ptch1^−^-pattern TFs, including Foxa2, Foxp1, Phf19, and Shh^−^Ptch1^+^-pattern TFs including Gli1, Gata2, Pbx1, Sox11 and Ctnnb1(Table S7). Except for Foxp1 whose target genes were directly obtained from the original paper, as the raw data were not available, we downloaded the ChIP-seq data for all other TFs and annotated the target genes using the same procedure as our own Gata3 ChIP-seq data analysis. In this network, only Shh^+^Ptch1^−^- and Shh^−^Ptch1^+^-pattern genes, as identified from our ISH data analysis, were included as potential target genes of the TFs. The regulatory relationships between TFs and target genes were based on the result of ChIP-seq data. Considering that many TFs can have both positive and negative regulatory functions, the TFs in one domain may target genes in the other domain as well. The complete gene regulatory network is illustrated in Figure S4A.

**Figure 5:**
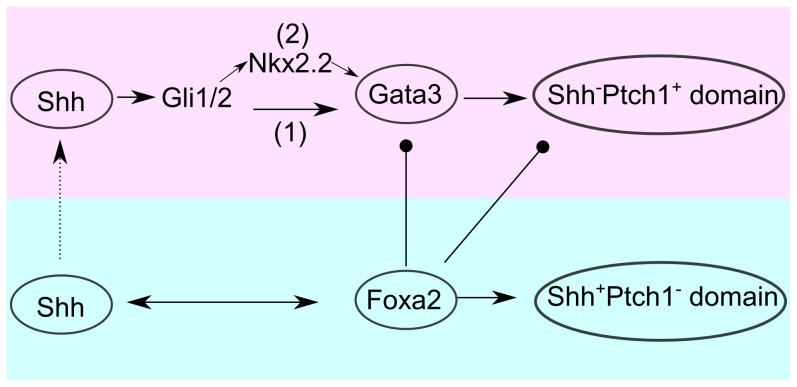
Model of Shh-mediated pattern formation in early mouse brain. Shh^+^Ptch1^−^ and Shh^−^Ptch1^+^ domains are colored by light red and light blue respectively. Broken line with triangular arrow indicates the diffusion of Shh. Solid line with triangular arrows represent up-regulation while circular arrows represent down-regulation. Two pathways of GLI regulating Gata3 may exist: direct regulation of Gata3 by Gli1/2 (pathway 1)was based on promoter analysis and indirect regulation of Gata3 by GLI through Nkx2.2 as proposed by Craven et al. [56].

As shown in this network, *Gata3* was targeted by TFs Foxa2 and Phf19. Since the expression of *Gata3* is mutually exclusive with *Foxa2* and *Phf19*, we propose that *Gata3* is negatively regulated by Foxa2 and Phfl9. Similarly to *Foxa2*, *Phf19* is expressed only in the floor plate of the entire hindbrain at the E11.5 stage. Studies showed that Phf19, a subunit of the polycomb repressor complex 2 (PRC2), has essential functions in cellular differentiation and embryonic development, in binding to H3K36me3 and being associated with H3k36me3 histone demethylase NO66, thereby mediating transcriptional silencing [49], [51]. We also found that *Gli2*, *Ptch1* and *Foxa2* are all targeted by Ctnnb1 while Foxa2 targets *Ctnnb1*. *Ctnnb1* encodes β-catenin, the signal transducer for the Wnt signaling pathway that is involved in early brain development [52]. Our analysis suggests that Gli2, Ptch1 and Foxa2 are downstream of this signaling pathway, reflecting its crosstalk with the Shh signaling pathway [53]. Using the MCODE program, we identified two gene regulatory modules in our network (Figure S4B and S4C). In the first module, *Dnmt3a*, *Nfasc* and *Mytl1* are co-regulated by both Gata3 and Pbx1 (Figure S4B). *Pbx1* is expressed throughout the entire alar plate and basal plate in the E11.5 mouse brain. It has been reported that embryos died at day E15/16 when *Pbx1* was deleted, with developmental defects in multiple organs [54]. In the second module, both Phf19 and Foxa2 target a total of 12 known genes that are enriched in the Shh^−^Ptch1^+^ domain, including *Ptch1*, while *Foxa2* is under the regulation of Phf19 and Foxa2 itself (Figure S4C). Furthermore, *Pax3* in the Shh^−^Ptch1^+^ domain is targeted by Foxa2 in the Shh^+^Ptch1^−^ domain, consistent with an earlier study showing that Pax3 inhibits the differentiation of the floor plate while Foxa2 itself activates its specification [55]. Previous studies have shown that Foxa2 positively regulates Shh^+^Ptch1^−^- pattern genes including *Shh*, *Foxa1* and *Ferd3l* while negatively regulating Shh^−^Ptch1^+^-pattern genes, including *Ptch1*, *Gli1* and *Gli2* [20]. Our study found that the targets of Foxa2 were not only enriched in the Shh^+^Ptch1^−^ domain but also in the Shh^−^Ptch1^+^ domain (Figure 3A). In particular, Foxa2 and Gata3 shared nine common target genes in the Shh^−^Ptch1^+^domain: *Abl1*, *Cadm1*, *Cotl1*, *Enc1*, *Foxn3*, *Isl2*, *Myt1l*, *Nfib* and *Sox12*. Therefore, Foxa2 not only up-regulates Shh^+^Ptch1^−^- pattern genes but also down-regulates Shh^−^Ptch1^+^-pattern genes and thereby antagonizes the effect of Gata3. This model is illustrated in Figure 5.

## Discussion

In this study, we integrated high-throughput ISH data, published microarray and ChIP-seq data, and our own experimental data, to investigate the gene regulatory circuit underlying the classical Shh signaling pathway in pattern formation of the E11.5 mouse brain. Based on our analysis, we propose that the fates of the adjacent Shh^+^Ptch1^−^ and Shh^−^Ptch1^+^ domains are determined by distinct sets of TFs that are up- or down-stream of the Shh signaling pathway. Among them, two pioneer factors, Gata3 and Foxa2, seem to play the key roles. Foxa2 up-regulates Shh^+^Ptch1^−^-pattern genes but down-regulates Shh^−^Ptch1^+^-pattern genes, while Gata3 up-regulates Shh^−^Ptch1^+^-pattern genes but has no obvious influence on Shh^+^Ptch1^−^-pattern genes. In our proposed model, the gene coding for Shh is activated by Foxa2 so that Shh is secreted in the Shh^+^Ptch1^−^ domain. However, the downstream effectors of Shh such as Gli1/2 are not activated in that domain due to inhibition by Foxa2. As Shh diffuses into the neighboring Shh^−^Ptch1^+^ domain, Gata3 is activated either directly by GLI family TFs or indirectly through Nkx2.2, as previously suggested [56] (Figure 5). Lending support to this hypothesis, it has been shown that the expression of Gata3 is increased upon Shh stimulation in 3T3-L1 cells [57]. Here we have reported that Gata3 can then turn on the gene regulatory programs to specify cell fates in the Shh^−^Ptch1^+^domain. During early mouse development, Foxa2 is already expressed in the CNS at least from E8.0 [58], one day earlier than the onset of Gata3 expression [11]. This temporal order is consistent with our model that Foxa2 is upstream of Gata3 in the Shh signaling cascade.

Recently, Shu et al. proposed a “seesaw” model in which counter-acting specifiers of two different lineages balance each other to maintain an undifferentiated cell state [59]. Loss of that balance leads to differentiation into one of the lineages. For example, Gata3, which is involved in mesendodermal specification, antagonizes Gmnn in ectodermal specification to induce pluripotency and facilitate reprogramming. In our study, we found an antagonizing relationship between Foxa2 and Gata3 and their competing roles in cell fate specification in their mutually exclusive domains: Shh^+^Ptch1^−^ and Shh^−^Ptch1^+^ domains in early brain development. Therefore, the spatial patterning along dorsal-ventral axis in early mouse brain can be due to the symmetry breaking of the balance between two counteracting forces represented by Foxa2 and Gata3. However, we think that Foxa2 and Gata3 are unlikely to be the only important specifiers for these two domains, as our reconstruction of the gene regulatory network implicated the involvement of a cohort of additional TFs. The detailed roles of these TFs and their relationships await future investigation, by both in vivo and in vitro experiments.

Currently, high-throughput ChIP-seq experiments to obtain direct regulatory interactions of TFs and their targets have been most conveniently conducted in in vitro systems. Typically, a ChIP-seq experiment requires around 10 million cells. This poses a technical challenge for conducting such ChIP experiments directly on the spatially restricted brain regions in embryos. To mimic midbrain dopaminergic neurons in the floor plate of the early mouse brain, Metzakopian et al. used the progenitors of midbrain dopaminergic neurons, formed by in vitro differentiation of *NestinLmx1a*-transfected ES cells, in their Foxa2 ChIP-seq experiments [20]. In our study, we used neuron-like PC12 cells as a surrogate for the Shh^−^Ptch1^+^ domain because of their high expression of Gata3 and ability to synthesize noradrenaline [35]. Our ChIP-seq experiments in PC12 cells uncovered new Gata3 target genes involved in neurotransmitter synthesis and release as well as axon guidance. Gata3 is also known for participating in the specification of serotonergic and noradrenergic neurons originated from the Shh^−^Ptch1^+^ domain. Our result showing that Gata3 targets *Slc18a1* and *Qdpr* adds new evidence for a central role of Gata3 in the development of serotonergic and noradrenergic neurons. Furthermore, we found that Gata3 regulates the SLIT/ROBO system. Interestingly, Metzakopian et al. have also found that Foxa2 regulates *Slit2* and *Slit3* in the floor plate region, analogous to the Shh^+^Ptch1^−^ domain in our study [20]. Our study thus sheds new lights on the potential parallel function of Gata3 to that of Foxa2 in axon guidance and neuron migration in the Shh^−^Ptch1^+^ domain [60]. Nevertheless, future in vivo experiments on embryonic Shh^−^Ptch1^+^ domain are necessary to validate our result in PC12 cells.

In addition to Shh, the genes coding for other signaling molecules such as Fgf and Bmp also show restricted spatial expression in ISH data. The combined expression patterns of these signaling molecules determine the specification of key neurons in the brain [42]. Using the same strategy as the one employed in this study, it should be possible to further delineate the gene regulatory networks controlled by these signaling pathways for distinct brain regions. Our study is the first example to systematically utilize high throughput ISH data to generate a new hypothesis of early brain development. This approach thus promises to be valuable in future work designed to unravel the molecular mechanisms that underlie spatial and temporal patterning during early animal development.

## Supporting Information

Figure S1 **The Shh^+^Ptch1^−^ and Shh^−^Ptch1^+^ domains.** The anatomical maps were downloaded from ABA and the domains were defined based on the expression patterns of *Shh* and *Ptch1* as shown in Figure 1.
(EPS)

Figure S2 **Heatmap of Shh^+^Ptch1^−^- and Shh^−^Ptch1^+^-pattern genes.** Blue and yellow colors represent the binary gene expression obtained from ABA as either expressed or non-expressed respectively.
(EPS)

Figure S3 **Shh^+^Ptch1^−^-/Shh^−^Ptch1^+^-pattern genes are significantly associated with early brain development.** (A) The overlap between Shh^+^Ptch1^−^-/Shh^−^Ptch1^+^-pattern genes and FP (floor plate) and VL (ventrolateral region) genes in E10.5 mouse brain from Gennet et al.'s study [33]. (B) The statistical significance of enrichment of shared genes between groups in (A). (C–D) Biological processes enriched in Shh^+^Ptch1^−^-pattern genes (C) and in Shh^−^Ptch1^+^-pattern genes (D).
(TIFF)

Figure S4 **Gene regulatory network of Shh^+^Ptch1^−^ and Shh^−^Ptch1^+^ domains reconstructed based on ChIP-seq data.** (A) The complete gene regulatory network consisting of nodes representing Shh^+^Ptch1^−^-pattern genes (red) and Shh^−^Ptch1^+^-pattern genes (blue) respectively. The edges with arrows represent the gene regulatory relationships from the TFs towards their target genes. The regulatory relationships (arrowed edges) starting from different TFs were indicated by different colors. Based on the KEGG reference pathway, red and blue stars mark molecules in the Shh signaling pathway and Wnt signaling pathway respectively. (B) and (C) are two regulatory modules identified by the Cytoscape plugin MCODE program in the complete network (see main text).
(TIFF)

Table S1 **Shh^+^Ptch1^−^-/Shh^−^Ptch1^+^-pattern genes and their enriched GO terms.**
(XLSX)

Table S2 **Motifs enriched in Shh^+^Ptch1^−^-/Shh^−^Ptch1^+^-pattern genes.**
(XLSX)

Table S3 **Results of our Real-time PCR assay.**
(XLSX)

Table S4 **Binding sites of Gata3 and functional annotation for Gata3 targets.**
(XLSX)

Table S5 **Genes up- or down-regulated by Shh.**
(XLSX)

Table S6 **Genes up- or down-regulated by Gata3.**
(XLSX)

Table S7 **ChIP-seq data used for constructing gene regulatory network in Shh^+^Ptch1^−^ and Shh^−^Ptch1^+^ domains.**
(XLSX)
