# Supplementary figures and images for: Reconstruction of the Gene Regulatory Network Involved in the Sonic Hedgehog Pathway with a Potential Role in Early Development of the Mouse Brain
Source: PLoS Comput Biol. 2014 Oct 9;10(10):e1003884. doi: 10.1371/journal.pcbi.1003884 (PMC4191885; doi:10.1371/journal.pcbi.1003884)

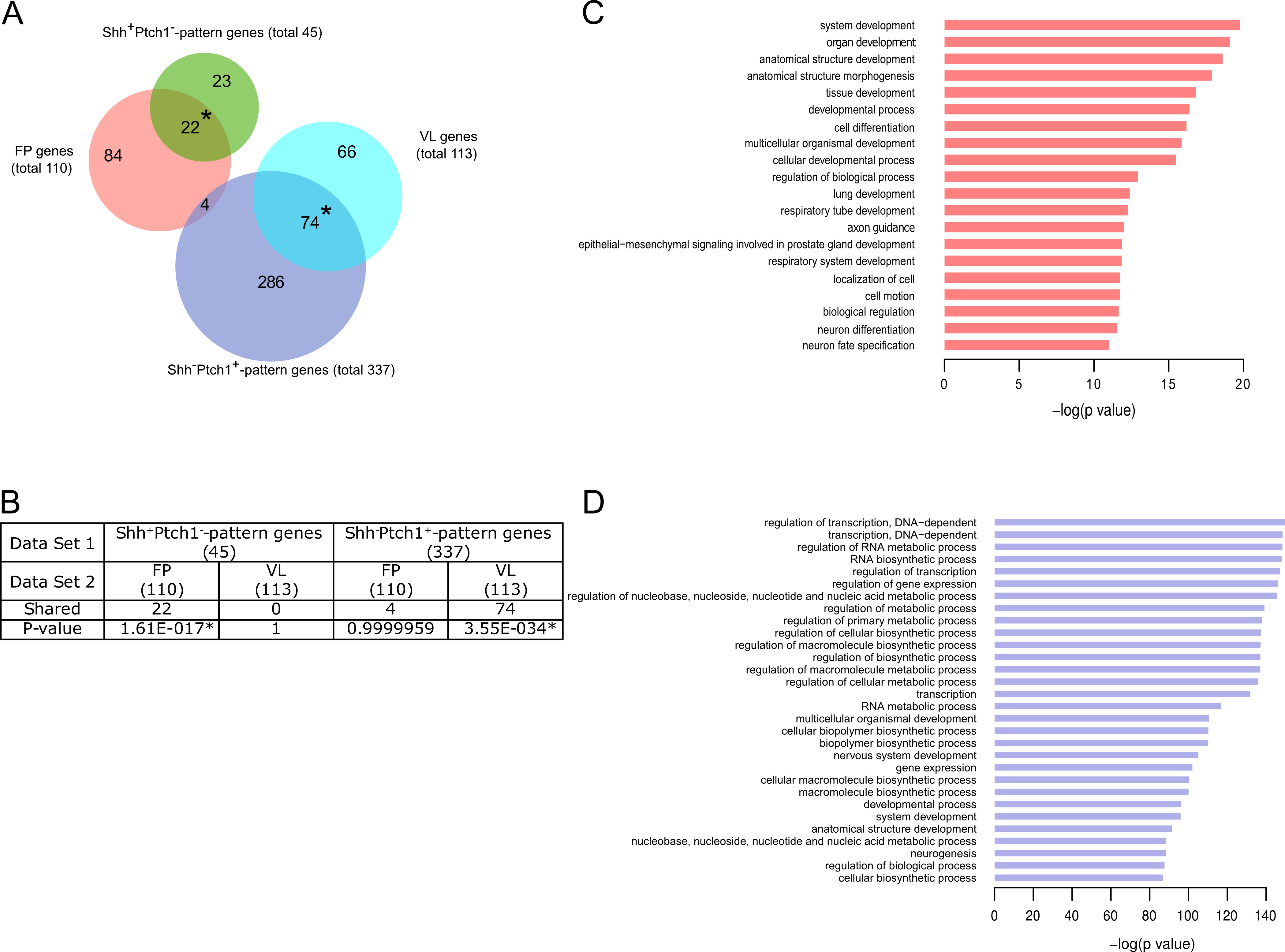

Supplement: Figure S3 — Shh+Ptch1−-/Shh−Ptch1+-pattern genes are significantly associated with early brain development. (A) The overlap between Shh+Ptch1−-/Shh−Ptch1+-pattern genes and FP (floor plate) and VL (ventrolateral region) genes in E10.5 mouse brain from Gennet et al.'s study [33]. (B) The statistical significance of enrichment of shared genes between groups in (A). (C–D) Biological processes enriched in Shh+Ptch1−-pattern genes (C) and in Shh−Ptch1+-pattern genes (D). (TIFF) [file pcbi.1003884.s003.tiff]

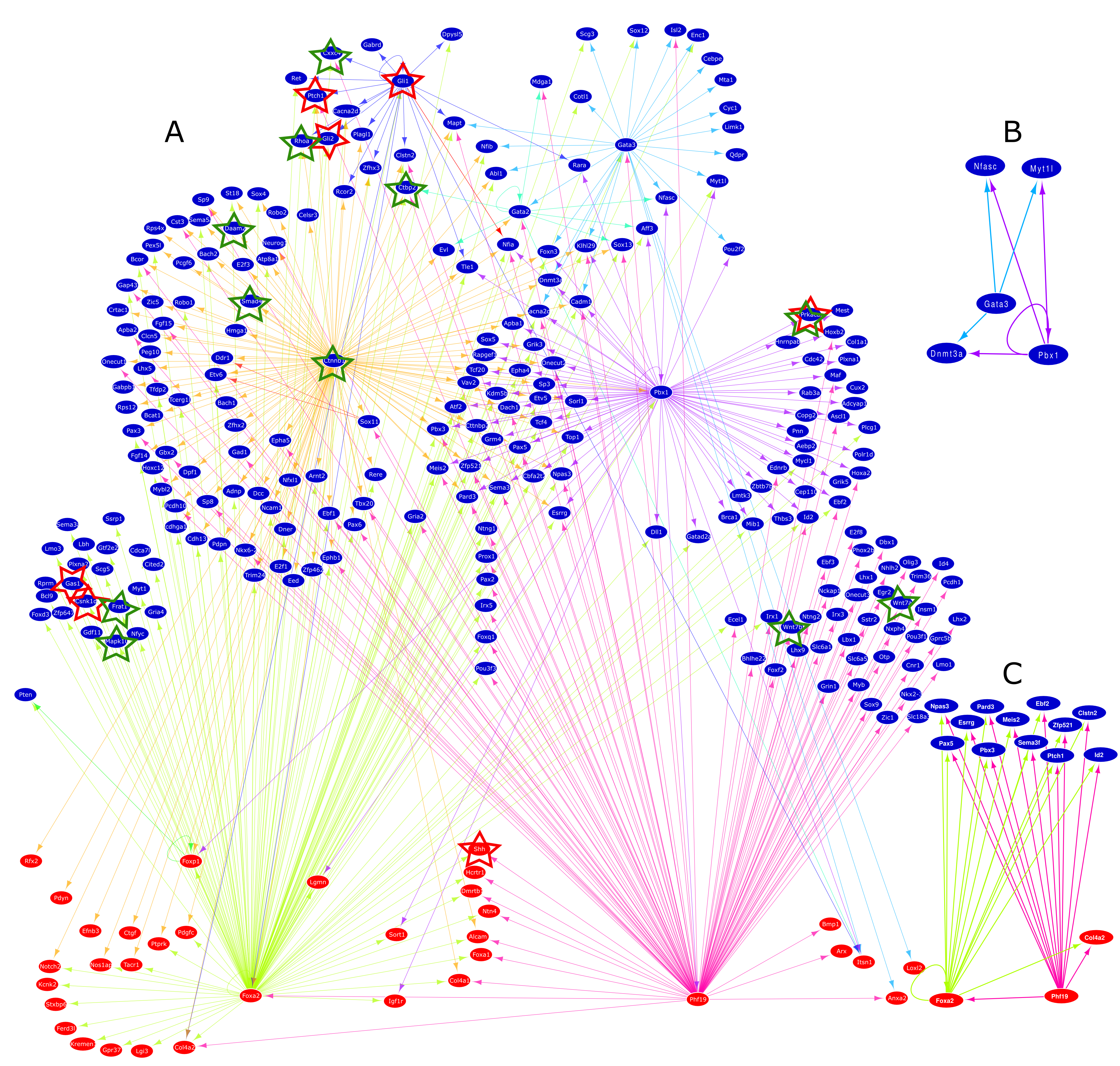

Supplement: Figure S4 — Gene regulatory network of Shh+Ptch1− and Shh−Ptch1+ domains reconstructed based on ChIP-seq data. (A) The complete gene regulatory network consisting of nodes representing Shh+Ptch1−-pattern genes (red) and Shh−Ptch1+-pattern genes (blue) respectively. The edges with arrows represent the gene regulatory relationships from the TFs towards their target genes. The regulatory relationships (arrowed edges) starting from different TFs were indicated by different colors. Based on the KEGG reference pathway, red and blue stars mark molecules in the Shh signaling pathway and Wnt signaling pathway respectively. (B) and (C) are two regulatory modules identified by the Cytoscape plugin MCODE program in the complete network (see main text). (TIFF) [file pcbi.1003884.s004.tiff]
